# 3D Reconstruction and Large-Scale Detection of Roads Based on UAV Imagery

**DOI:** 10.3390/ma18092133

**Published:** 2025-05-06

**Authors:** Xiang Zhang, Shuwei Cheng, Pu’an Wang, Hao Zheng, Xu Yang, Yaolin Guo

**Affiliations:** 1 Yunnan Transportation Science Research Institute Co., Ltd., Kunming 650011, China; yhgszx123@163.com (X.Z.); yhgscsw123@163.com (S.C.); 13708711876@163.com (P.W.); 2 School of Highway, Chang’an University, Xi’an 710064, China; yang.xu@chd.edu.cn; 3 School of Future Transportation, Chang’an University, Xi’an 710064, China; 2020900071@chd.edu.cn

**Keywords:** UAV, 3D reconstruction, point cloud, disease detection, road health assessment

## Abstract

Accurate and efficient detection of road damage is crucial in traffic safety and maintenance management. Traditional road detection methods have problems such as low efficiency and insufficient accuracy, making it difficult to meet the needs of large-scale road health assessments. With the development of drone technology and computer vision, new ideas have been provided for the automatic detection of road diseases. The existing drone-based road detection methods have poor performance in dealing with complex road scenes such as vehicle occlusion, and there is still room for improvement in 3D modeling accuracy and disease detection accuracy, lacking a comprehensive and efficient solution. This paper proposes a UAV (Unmanned Aerial Vehicle)-based 3D reconstruction and large-scale disease detection method for roads. By capturing aerial images with UAVs and utilizing an improved YOLOv8 model, vehicles in the images are identified and removed. Apply MVSNet (Multi-View Stereo Network) 3D reconstruction algorithm for road surface modeling, and finally use point cloud processing and DBSCAN (Density-Based Spatial Clustering of Applications with Noise) clustering for disease detection. The experimental results show that this method performs excellently in terms of 3D modeling accuracy and speed. Compared with the traditional colmap method, the reconstruction speed is greatly improved, and the reconstruction density is three times that of colmap. Meanwhile, the reconstructed point cloud can effectively detect road smoothness and settlement. This study provides a new method for effective disease detection under complex road conditions, suitable for large-scale road health assessment tasks.

## 1. Introduction

### 1.1. Research Background and Significance

Road facilities are the core components of the transportation infrastructure system, and the stable operation of road infrastructure depends on timely and effective disease diagnosis and maintenance [1]. Road diseases (such as cracks, potholes, etc.) are often caused by natural factors and traffic load. If not detected and repaired in time, it may lead to serious safety hazards and increase maintenance costs [2]. Therefore, it is necessary to automatically identify all kinds of diseases and extract information efficiently to judge maintenance opportunities and select maintenance strategies [3].

The manual field investigation method used for a long time in the past has problems such as low efficiency, strong subjectivity, and poor safety, and it can no longer meet the needs of current pavement disease detection [4]. At present, the commonly used road disease detection methods mainly focus on the automatic recording and analysis of disease information by using specially equipped sensor detection vehicles driving along the road [5]. However, its driving speed is still restricted by road conditions and traffic conditions, and it cannot quickly cover a large area [6]. In addition, large-scale diseases such as rutting and settlement of asphalt pavement cannot be well recognized [7].

In recent years, UAV-based detection methods have attracted wide attention, with advantages such as high coverage and strong flexibility, and can be combined with deep learning technology to improve detection efficiency and accuracy [8], providing strong support for modern transportation infrastructure management. Rapid deployment of UAVs can achieve efficient coverage of a large area of road network and shorten the response time of disease detection [9]. High-resolution sensors collect detailed road image data and combine deep learning algorithms to identify and classify various types of road disorders and facilities (such as rutting, flatness, large-scale settlement, marking, signage, etc.) [10].

With the development of 3D measurement technology, various 3D imaging systems have been used for pavement data acquisition, including time-of-flight [11,12], projection laser system [13], and stereo vision system [14,15,16]. However, the performance of these systems varies in terms of imaging accuracy, resolution, speed, and field of view (FOV) [17,18].

To sum up, it is of great significance to apply deep learning and 3D imaging technology to UAV road detection. This study aims to develop an efficient road 3D modeling and large-scale disease detection system by combining UAV and deep learning technology to improve the accuracy and real-time road disease detection. With the help of the improved YOLOv8 model, the road surface image is recognized quickly, and MVSNet technology is used to complete the three-dimensional reconstruction of the road. Combined with point cloud processing and the DBSCAN clustering algorithm, this study can effectively detect a wide range of road surface diseases, such as rutting and uneven settlement. The system is not only suitable for disease detection under complex road conditions but also provides an innovative solution for road health assessment, meeting the needs of modern road maintenance management for intelligence and automation [19].

### 1.2. Research Status at Home and Abroad

Pavement distress detection has consistently been a focal point of research within the field of road engineering. The automated detection of visible distresses, such as pavement cracks, potholes, and deformations, has attracted considerable research interest. Due to the maintenance and management demands arising from early large-scale roadway network construction, developed countries such as the United States, Canada, Australia, and Japan have pioneered advancements in automated pavement distress detection, leading to the development of numerous pavement data collection devices and automated recognition methods [19,20,21,22]. As early as 2004, the NCHRP-334 report summarized methods for the automated collection and processing of pavement condition data, encompassing pavement damage identification, rut depth measurement, and ride quality assessment. This report comprehensively encapsulated the research progress and practical cases of early pavement distress detection [23]. With the continuous advancement of sensor technology, various commercial pavement inspection systems are now capable of multidimensional data collection concerning pavement surface characteristics, and intelligent recognition of pavement distresses based on different data types has emerged as a new research direction.

In recent years, pavement inspection systems based on three-dimensional laser scanning technology have increasingly been employed for pavement damage and texture assessment, producing high-quality 3D pavement images. The team led by Kelvin Wang [24] at Oklahoma State University has developed a pavement data collection vehicle equipped with the PaveVision3D high-resolution 3D imaging system, which enables the rapid collection of millimeter-level three-dimensional data for lanes. Commercial multifunctional inspection systems, such as PSI’s PathRunner, Canada’s ARNA 9000 [25], and ZOYON-RTM from Wuhan University of Technology [7], have all integrated laser 3D imaging modules, which can generate high-precision laser point cloud depth maps, achieving collection speeds of up to 100 km/h.

Two-dimensional color images and three-dimensional depth images serve as the primary data sources for current pavement distress identification. Two-dimensional image recognition techniques primarily rely on visual color differences to distinguish distressed areas. Typically, damaged regions, such as cracks and potholes, appear darker compared to normal pavement, enabling distress identification through pixel variations. In contrast, three-dimensional image recognition methods predominantly employ laser scanning and stereo imaging to generate depth images, identifying distresses based on the height characteristics of the distress areas, which are generally significantly higher or lower than the pavement surface. Currently, most research efforts focus on the automated identification of pavement distresses based on two-dimensional images, which can be broadly categorized into image processing-based recognition methods and deep learning-based recognition methods. While two-dimensional images offer the advantage of ease of acquisition, they are susceptible to interference from factors such as lighting, color, and pavement markings, posing substantial analytical challenges in complex environments. Xu Zhigang [26], in summarizing the latest developments in pavement damage detection systems and image recognition algorithms, pointed out that although deep learning methods can enhance the generalization of distress recognition, algorithms based on two-dimensional distress images struggle to achieve an optimal balance among accuracy, robustness, and real-time performance. Furthermore, the advancement of industrial three-dimensional imaging technology has provided various pathways for the digitization of pavement distresses. Mathavan [17] reviewed the applicability of various three-dimensional imaging technologies across different contexts, highlighting their significant potential for application in pavement distress detection and measurement. Researchers such as Cao [16] and Yang [27] have summarized the progress in the automatic recognition of three-dimensional distress images, emphasizing that the integration of three-dimensional imaging and deep learning can notably enhance the robustness of distress identification. It is anticipated that the convergence of three-dimensional imaging technology and deep learning methodologies for the intelligent diagnosis of pavement distress at different scales will become a key focus in future research.

In light of the current research trends both domestically and internationally, as well as a preliminary assessment of future research focuses, it has been decided to utilize Unmanned Aerial Vehicles to assist in the identification and detection of large-scale pavement distresses. Unmanned Aerial Vehicle photography platforms possess the capability to efficiently capture pavement images, enabling the three-dimensional reconstruction of large-scale scenes. To evaluate the spatial accuracy of pavement models generated by the UAV Structure-from-Motion (UAV-SfM) stereo vision system, some studies have utilized Ground Control Points (GCP) and terrestrial laser scanning to estimate the error distribution of the generated point clouds [28,29]. Based on the pavement point cloud models generated by UAV-SfM, Inzerillo et al. [30] measured the depth and width of cracks in asphalt pavements, while Roberts et al. [5] analyzed the deformation severity of the pavement surface using digital models. Furthermore, similar UAV-SfM workflows have been utilized to efficiently measure the International Roughness Index (IRI) of pavements and potholes [31,32]. To determine the optimal flight altitude for drones, Saad [10] and Romero-Chambi [6] quantified the impact of flight height on the three-dimensional reconstruction of potholes, discovering that low-altitude flights could achieve high-precision distress measurements. Although the UAV-SfM stereo vision system can efficiently reconstruct entire pavement scenes, the ongoing traffic from vehicles on the road has limited current research and applications, primarily to closed rosad environments with minimal traffic [10,30,31].

## 2. UAV Data Acquisition

### 2.1. UAV Flight Platform and Flight Parameters

**Figure 1:**
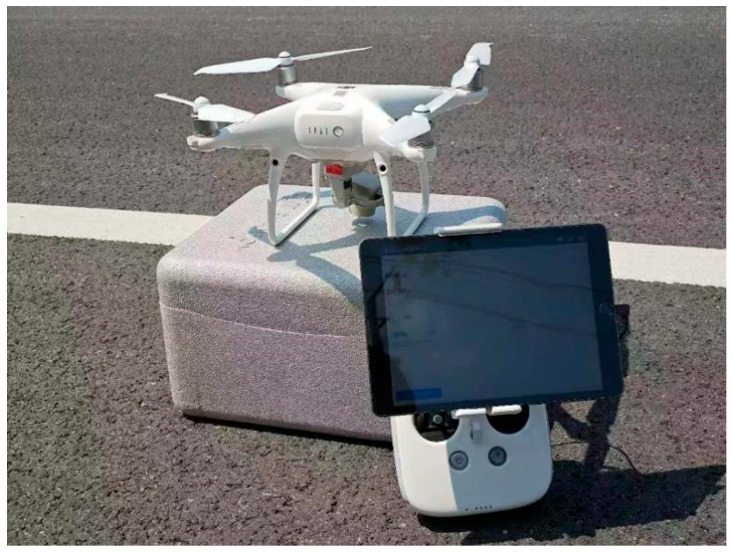
UAV flight acquisition platform.

The UAV used in this experiment is the DJI Phantom4 Pro V2.0 (Chinese DJI brand) (in Figure 1). Equipped with a flight remote control, the maximum control distance is 6 km, the maximum flight height is 500 m, and the duration of a single flight can reach 25 to 30 min. At the same time, it is equipped with a high-resolution camera, which has high spatial accuracy and image clarity. Table 1 shows the detailed equipment parameters and flight parameters.

It should be noted that the quality of 3D reconstruction based on UAV photography is affected by many factors, such as flight height, image overlap rate, and environmental noise, and photography parameters should be reasonably set according to different scenes. In dynamic traffic scenes, vehicles running on the road will block the road surface, so improving the image overlap rate is necessary to avoid completely blocking the road surface. The image overlap rate will also affect the quality of 3D model reconstruction. Typically, the image overlap rate is set to 75%, the flight height of the UAV along the road is set to 15 m, and the flight speed to 2 m/s to enhance the accuracy of point cloud reconstruction.

### 2.2. Data Acquisition

The data collection area is on Fuyao Road, Fuping County, Shaanxi Province. The road runs across Fuping County from east to west, with a total length of 41.4 km. The two ends of Fuyao Road are connected to the expressway, and the freight demand of the surrounding industries leads to the daily traffic of cargo vehicles on the test road, which are mainly medium and heavy trucks, resulting in frequent road diseases. Based on this, we selected the road sections with large traffic flow and serious diseases for collection through field survey. To encompass a variety of road diseases., this test road was collected in five sections, with a total length of about 5 km. The specific division is shown in Table 2.

**Figure 2:**
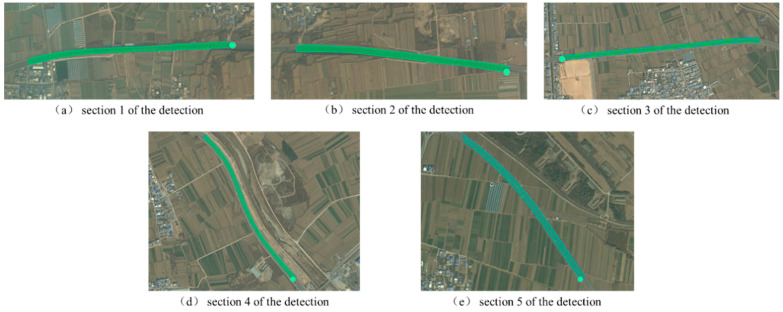
Route planning map for different detection sections.

In the process of acquisition, the waypoint control software DJI GS Pro (GS Pro2.0) is used to plan the mapping task of UAVs. According to the specified flight and photography parameters, different routes and waypoints are automatically generated in the detection area. Figure 2 shows the route planning diagram of the detection section. After the route planning is completed, the UAV takes off from both sides of the road. The lens angle is perpendicular to the lane, flies according to the predetermined flight path and waypoint, automatically collects the road surface image at the same time, and adjusts the flight height and speed in real time according to the actual situation of the road.

## 3. Methods

### 3.1. Three-Dimensional Road Modeling Based on UAV Imagery

#### 3.1.1. Vehicle Positioning and Removal Based on YOLOV8 Network Algorithm

Since the vehicle will block the road surface in the photos collected by UAV, it needs to be identified and removed from the aerial image to improve the accuracy and efficiency of 3D reconstruction. Compared to various object detection deep learning algorithms, the YOLO network is widely used because it offers the best trade-off between accuracy and speed.

**Figure 3:**
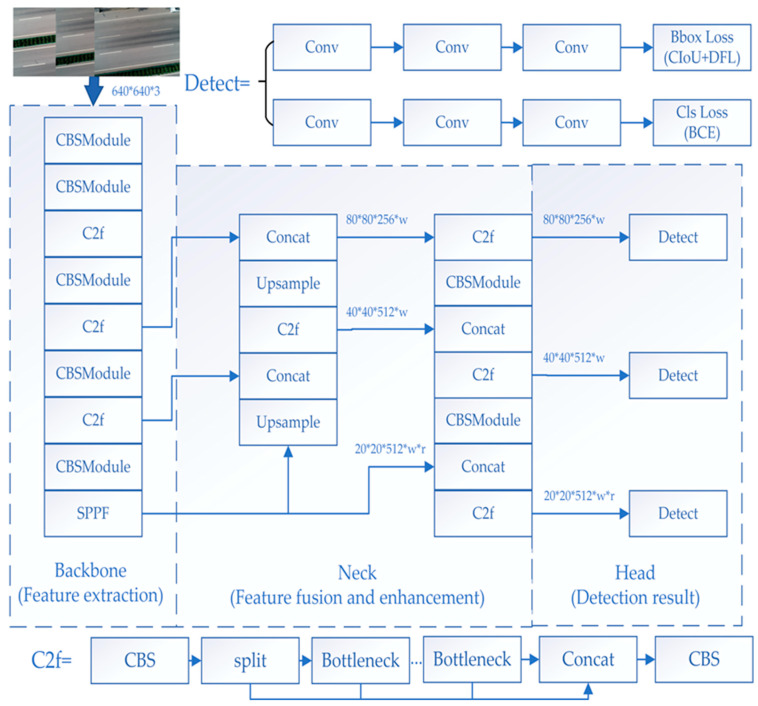
YOLOv8 detection network architecture diagram.

YOLOv8 inherits the consistent feature of the YOLO series—an end-to-end object detection framework that generates target bounding boxes and category labels directly from the input image, so the YOLOV8 detector is used to identify vehicles in UAV images. Figure 3 shows the network architecture of YOLOv8.

In this experiment, the Fuyao Road data set collected by UAV was selected for self-collected datasets. Firstly, the image was processed into 640 × 640 size, and the data set was further divided into training set, test set, and verification set, with a ratio of 8:1:1, to support the effective training and performance evaluation of the model.

In the training process, the validation set is used to evaluate the model performance, and the convergence of the model’s loss function and other indicators such as Precision, Recall, and mean average accuracy (mAP) are checked. These basic assessment measures can be estimated based on True Positive (TP), True Negative (TN), False Positive (FP), and False Negative (FN). mAP is used to evaluate multi-classification target recognition tasks, while AP is used to evaluate the accuracy of single target recognition tasks. Therefore, AP is used in this paper to evaluate the vehicle recognition accuracy of aerial photography images. After the training is completed, the test set is used to conduct independent tests to evaluate the generalization ability and performance of the model, especially the accuracy of vehicle detection under different flight heights, angles, and lighting conditions of the UAV. The Precision and Recall can be defined as follows:

(1) $$Precision=\frac{TP}{TP+FP}$$

(2) $$Recall=\frac{TP}{TP+FN}$$

where TP is the number of correctly detected vehicles, FP is other objects incorrectly detected as vehicles, and FN is the number of undetected vehicles. The AP and mAP can be defined as follows:

(3) $$AP=\int_{0}^{1}Precision(R)dR$$

(4) $$mAP=\frac{\sum_{i=0}^{n}{AP}_{\left(i\right)}}{n}$$

In the evaluation of the target recognition model, the TP value is affected by the IoU (Intersection over Union) threshold, and the accuracy of the prediction boundary box is determined by whether the IoU value between the prediction box and the truth box is greater than the preset threshold. Therefore, Pr, Re, and AP also depend on the choice of IoU thresholds. In this experiment, the IoU threshold was set at 0.5 to evaluate the vehicle recognition results.

**Figure 4:**
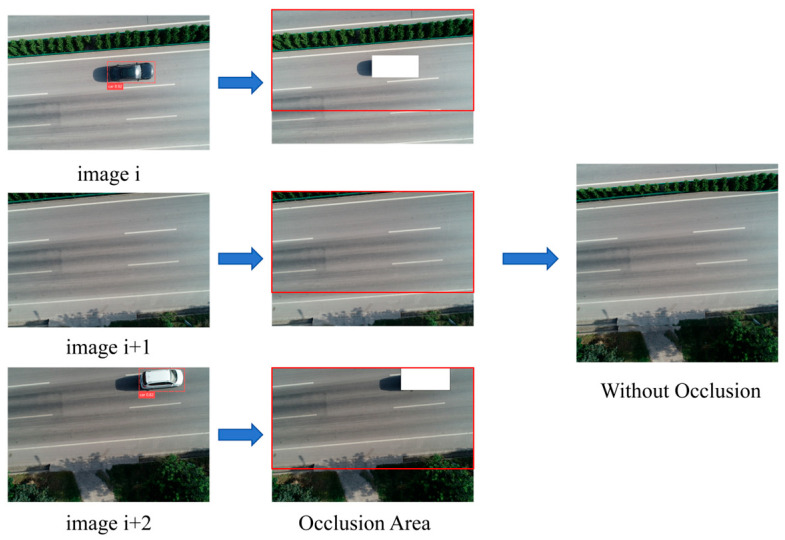
Road occluded area elimination.

The location of the vehicle in the image was identified by the YOLOv8 detector, and the detection box of the occluded area was replaced with a white block with pixel 0. In this experiment, to eliminate the occluded area of the road surface, UAV shooting technology was used to take multiple shots of the overlapping area. Even if the road surface in one image is partially obscured by vehicles, the obscured area can still be seen in other images. The overlap rate of UAV photography is set at 75%. Once the occluded area in each image is identified, the pavement occluded area can be subtracted from the overlapping area of the two adjacent images to estimate the actual situation of the road surface, as shown in Figure 4.

#### 3.1.2. Three-Dimensional Road Surface Reconstruction Based on MVSNet

**Figure 5:**
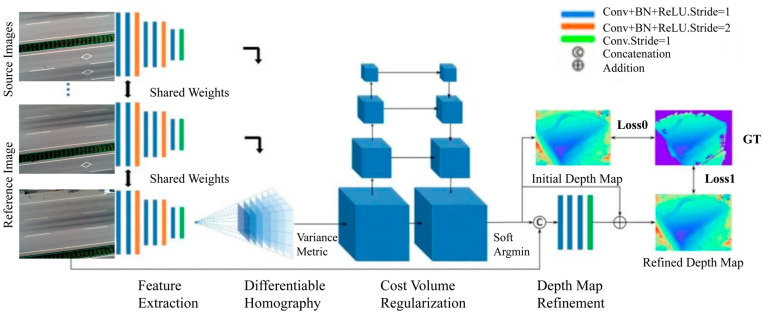
MVSNet network architecture.

MVSNet is a depth estimation network based on deep learning. This network performs convolution learning on input 2D images from multiple perspectives through convolutional layers, extracts image features, and obtains feature maps containing image texture, shape, depth, and other information. Then, based on the generated feature maps and input camera position and pose information, The 3D cost body is constructed by homography warping, and then the obtained cost body is regularized to obtain the final probability body. Depth estimation and optimization are carried out from the probability body to generate the depth map of the image. Finally, the depth map is merged to generate a point cloud model to complete the reconstruction of the target scene. The network architecture of MVSNet is shown in Figure 5.

MVSNet 3D reconstruction requires the resolution of the input image to be 1200 × 1600. Therefore, the 3:4 ratio is used to take photos when collecting the pavement data set to avoid image distortion caused by subsequent image resolution processing. After the image with the appropriate pixel size is processed, colmap is used for preliminary preprocessing of both the training data set and test data set to obtain the camera pose and internal parameter information corresponding to each image.

For the training data set, after preliminary processing, colmap is used for depth estimation and the corresponding depth map of each image of the target scene is obtained.

Compared with the test data set, the processing of the training data set mainly involves the process of generating depth map files, which is mainly used for deep learning prediction of images in the model training process.

Under different environmental conditions, images can affect the quality of reconstruction. For instance, uneven lighting may cause local overexposure or underexposure in the image, which can affect the extraction and matching of feature points and, ultimately, the accuracy of 3D reconstruction. In low-light conditions, the image quality of the camera deteriorates, and noise increases. This makes it more difficult to accurately identify feature points during image matching, thereby affecting the accuracy and completeness of the reconstruction results. Therefore, in complex environmental conditions such as cloudy or rainy days, the model needs to undergo data augmentation to ensure its stability and accuracy under different weather and lighting conditions, thereby improving its generalization ability in these conditions.

**Figure 6:**
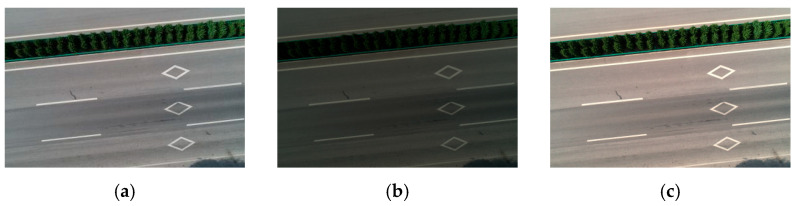
Data enhancement: (**a**) master drawing, (**b**) simulated overcast, (**c**) simulated sunny day.

By adjusting the brightness of the pictures in the training data set, the picture scenes under different weather conditions, such as sunny and cloudy days, are simulated. This simulation method can improve the generalization of the training model for scene reconstruction under different light scenes so that the model can more accurately reconstruct the real scene when facing various light conditions in the actual application scene. Figure 6 displays the target scene image and its simulated images under different weather conditions.

After the training data set is made, the model is trained in the deployed MVSNet environment. When the loss value reaches the threshold, the training stops automatically. Through further analysis and testing, the performance of MVSNet in practical pavement application scenarios can be more deeply understood and the model can be further optimized.

### 3.2. Disease Detection Technology Based on Multi-Scale and Full-View

#### 3.2.1. Data Processing

Based on the above 3D reconstruction, the road surface point cloud plane is not parallel to the axis x and y plane, which affects the subsequent extraction of disease information based on coordinates. Therefore, it is necessary to carry out the coordinate rotation for the reconstructed 3D model. The reconstructed three-dimensional model of the road table contains the disease information of multiple lanes. For each lane, the vehicle load is concentrated on the wheel path. As a result, lateral and longitudinal surface deformations are symmetrically distributed on the lane. To measure rutting and flatness, lane division is necessary.

**Figure 7:**
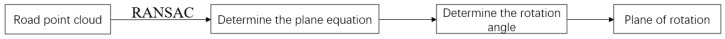
Coordinate rotation process.

Before the rotation operation is performed, the plane coordinates of the road surface are determined first. In this study, the plane coordinates of the road surface are iterated by the RANSAC (Random Sample Consensus) algorithm, and then the normal vector of the road surface is determined. Figure 7 shows the coordinate rotation process.

As a basic road sign, lane lines are often used to define the trajectory of vehicles. In this study, road markings are extracted by intelligent extraction of lane demarcation lines, and then lane division is carried out.

**Figure 8:**
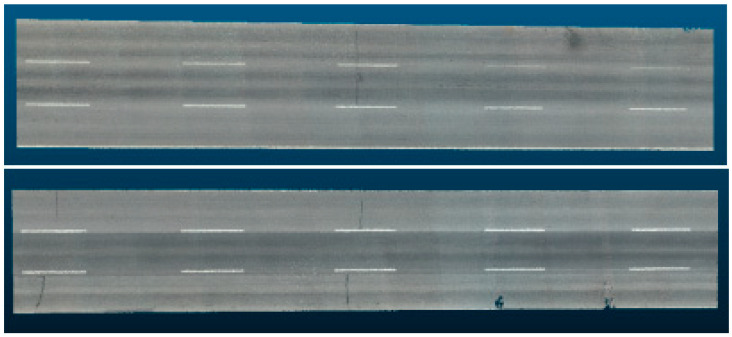
Extracted lane point cloud image.

First, the point cloud data of the multi-vehicle road table are loaded into the processing system, which contains the spatial coordinates (X, Y, Z) of each point, as well as color information. According to the color information, set the color threshold and screen out the points close to white, which is the extracted lane line point cloud. The noise removal process based on a statistical filter is carried out to reduce the subsequent fitting error. After the denoised mark line point cloud is obtained, the linear fitting based on the least square method is carried out to determine the mark line linear equation. According to the linear equation obtained, the threshold value is set for the whole road surface point cloud data for segmentation, and the lane point cloud is determined for lane division. Figure 8 shows the point cloud image after lane segmentation.

#### 3.2.2. Calculation of Road Roughness Based on Vehicle Road Model

Firstly, the lane area is located through lane line segmentation and fitting, and the profile plane extraction line is set according to the curvature of the lane line in the lane according to a certain transverse interval distance. To cover the profile data of the whole lane and the smoothness evaluation, the longitudinal profiles and elevation values of 1/4 and 3/4 transverse positions of each lane were extracted at fixed intervals for a single lane, which was used to calculate the international smoothness values of multiple transverse positions in subsequent models.

The evaluation index of road roughness is mainly based on the road-suspension response model. The road roughness is characterized by the vertical vibration response generated by the vehicle suspension moving on the road. The vehicle suspension model is the core of flatness evaluation. The degree of freedom of the suspension model can be divided into the two-degrees-of-freedom quarter car model, a four-degrees-of-freedom half-car model, and a seven-degrees-of-freedom whole-vehicle model. Among them, the International Roughness Index (IRI) is the most commonly used pavement roughness evaluation index. This index is a response evaluation index proposed by the World Bank to measure the driving quality of infrastructure in a unified way. Its core is to estimate the dynamic response of vehicle suspension relative to road profile by using a two-degrees-of-freedom quarter car model.

**Figure 9:**
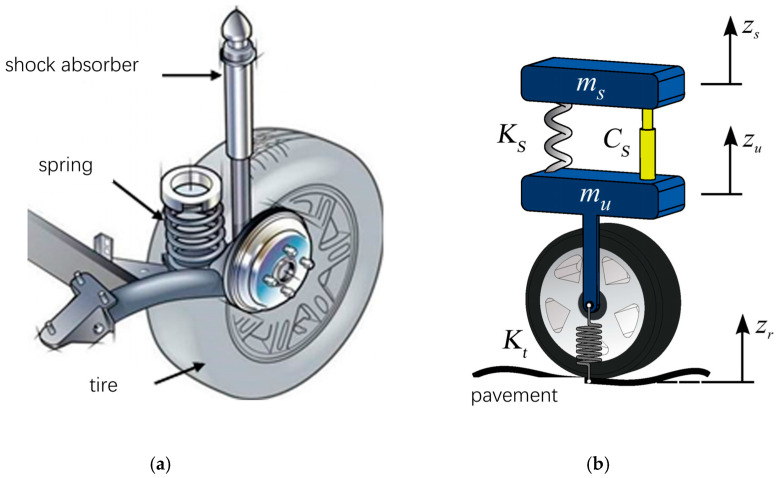
Quarter car model: (**a**) vehicle suspension structure, (**b**) quarter car model.

As shown in Figure 9, the vehicle suspension system includes auxiliary mechanisms such as springs, damping shock absorbers, swing arms, and tires. The mathematical model of a quarter car suspension is composed of a tire, a spring with stiffness $K_{s\;}$ and a shock absorber with damping $C_{s}$, in which the unsprung mass is $m_{s}$ and the unsprung mass is $m_{u}$. The system assumes that the tire is always in contact with the road, the tire stiffness is $K_{t}$ l, and the vehicle speed is fixed at 80 km/h. According to the one-quarter car model, IRI can be calculated by the following formula:

(5) $$IRI=\frac{1}{L}\int_{0}^{\frac{L}{V}}\left|{\dot{Z}}_{s}-{\dot{Z}}_{u}\right|dt$$

(6) $$IRI=\frac{1}{L}\int_{0}^{L}\left|Z_{s}-Z_{u}\right|dx$$

where L is the length of the road profile; V is the speed of the vehicle; ${\dot{Z}}_{s}$ is the vertical velocity of the sprung mass part; $Z_{s}$ is the vertical movement distance of the spring-mass part; ${\dot{Z}}_{u}$ is the vertical velocity of the unsprung mass part; $Z_{u}$ is the vertical movement distance of the unsprung mass part; dt is the time differential; and dx is the distance differential.

The essence of the IRI calculation formula above is that when the vehicle travels along the road profile curve at a certain speed, the suspension will vibrate up and down under the excitation of the uneven road surface. Therefore, the cumulative motion trajectory of the vehicle suspension relative to the vehicle body is calculated to reflect the road surface flatness. For ease of solution, the formula can be simplified as follows:

(7) $$IRI=\frac{1}{L}\int_{0}^{L}\left|Z_{s}-Z_{u}\right|dx$$

where n is the sample number of test points.

According to the above discrete calculation formula, IRI value calculation must obtain the vertical distance difference between each measuring point’s upper and lower parts. In the field physical measurement process, the motion distance of the two parts can be measured by installing sensors on the bottom of the suspension and the body of the test car. In the process of digital simulation, these two parameters need to be solved using the road profile curve and suspension dynamic equation. With the upper part of the suspension spring and the lower part of the spring divided into the center, the following second-order vibration differential equation can be established.

(8) $$m_{s}{\ddot{Z}}_{s}=K_{s}\left(Z_{u}-Z_{s}\right)+C_{s}\left({\dot{Z}}_{u}-{\dot{Z}}_{s}\right)$$

(9) $$m_{u}{\ddot{Z}}_{u}={-K}_{s}\left(Z_{u}-Z_{s}\right)-C_{s}\left({\dot{Z}}_{u}-{\dot{Z}}_{s}\right)+K_{t}\left(Z_{r}-Z_{u}\right)$$

where ${\ddot{Z}}_{s}$ is the vertical acceleration of the spring part; ${\ddot{Z}}_{u}$ is the vertical acceleration of the unsprung part; and $Z_{r}$ is the vertical change distance of the pavement profile.

According to the standard suspension parameters of the quarter car, u = m_u_/m_s_ = 0.15, C = C_s_/m_s_ = 6.00 s^−1^, K_1_ = K_s_/m_s_ = 653 s^−2^, K_2_ = K_t_/m_s_ = 63.3 s^−2^. The above Formulas (10) and (11) can be simplified as follows:

(10) $${\ddot{Z}}_{s}=K_{1}\left(Z_{u}-Z_{s}\right)+C\left({\dot{Z}}_{u}-{\dot{Z}}_{s}\right)$$

(11) $${\ddot{Z}}_{u}=\frac{K_{1}}{\mu }\left(Z_{s}-Z_{u}\right)+\frac{C}{\mu }\left({\dot{Z}}_{s}-{\dot{Z}}_{u}\right)+\frac{K_{2}}{\mu }\left(Z_{r}-Z_{u}\right)$$

The solution of the above equation depends on calculating $\left[{\dot{Z}}_{s}{\ddot{Z}}_{s}{\dot{Z}}_{u}{\ddot{Z}}_{u}\right]$ these four state variables, $Z\left(t\right)={\left[Z_{s}{\dot{Z}}_{s}Z_{u}{\dot{Z}}_{u}\right]}^{T}$, which can be converted to the following:

(12) $$\frac{dZ\left(t\right)}{dt}=AZ\left(t\right)+BZ_{r}\left(t\right)$$

(13) $$A=\left[\begin{array}{cc} \begin{array}{cc} 0 & 1\\ -K_{1} & -C \end{array} & \begin{array}{cc} \;\;\;\;0\;\;\;\;\;\; & \;\;\;\;0\\ {\;\;\;\;K}_{1}\;\;\;\;\; & \;\;\;\;C \end{array}\\ \begin{array}{cc} 0 & 0\\ \frac{K_{1}}{\mu } & \frac{C}{\mu } \end{array} & \begin{array}{cc} \;\;\;\;0 & 1\\ -\frac{K_{1}+K_{2}}{\mu } & -\frac{C}{\mu }\;\;\;\; \end{array} \end{array}\right]$$

(14) $$B=\left[\begin{array}{c} \begin{array}{c} 0\\ 0 \end{array}\\ \begin{array}{c} 0\\ \frac{K_{2}}{\mu } \end{array} \end{array}\right]$$

Formula (11) is a non-homogeneous differential equation. According to the initial value of $Z\left(t\right)$ and the gradient of the section curve, $Z\left(t\right)$ at any time can be solved by recursion, and the motion of $Z_{s}$ and $Z_{u}$ at each measuring point can be obtained to calculate IRI.

#### 3.2.3. Pavement Abnormal Deformation Recognition Based on DBSCAN Clustering

In addition to the two common deformation forms of transverse rutting deformation and longitudinal pavement deformation, large-scale abnormal deformation also exists on some pavement, which is mainly caused by factors such as uneven settlement of subgrade, insufficient compaction degree, and shrinkage and expansion of materials. It is difficult to identify large-scale abnormal deformation of pavement because of its random distribution, fuzzy boundary, and complex shape. Limited by the spatial distribution range and complex change characteristics of large-scale abnormal deformation, most current studies reflect the abnormal deformation through the multi-angle one-dimensional cross-section. However, to fully measure the three-dimensional shape of large-scale abnormal deformation, it is necessary to extract the two-dimensional boundary of the disease from the three-dimensional digital elevation map. Therefore, considering the complex form and fuzzy boundary of large-scale abnormal deformation, this paper adopts unsupervised three-dimensional elevation clustering to extract spatial elevation demarcation points and identify abnormal deformation regions.

**Figure 10:**
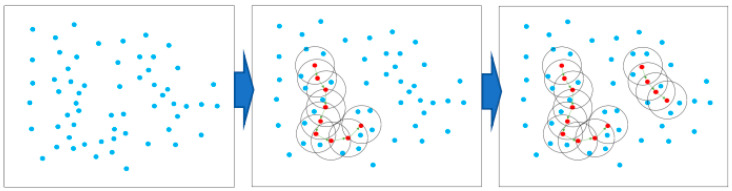
DBSCAN algorithm clustering principle diagram.

The DBSCAN algorithm identifies clusters by density. Its core principle is to divide data into core points, boundary points, and noise points according to the density of data points. First, the algorithm selects a distance threshold (ε) and a minimum number of neighbor points (MinPts) and then starts from any unvisited core point and identifies points in its neighborhood. If the number of points in the neighborhood exceeds MinPts, the points are grouped into the same cluster, and the cluster is recursively expanded until no more points can be added. Finally, the resulting clusters have arbitrary shapes and can effectively identify noisy data. The overall search process is shown in Figure 10.

The input to the DBSCAN cluster is multidimensional data and is suitable for point cloud data with different densities. Its main goal is to cluster data points based on their density characteristics, which is particularly suitable for discrete, non-spherical spatial distributions. In the clustering process of DBSCAN, you first need to define two important parameters: ε (neighborhood radius) and MinPts (minimum number of points in the neighborhood).

Initial step: In three-dimensional space, each observed data point is first classified according to the given ε and MinPts conditions. For each point p, calculate the number of points in its ε neighborhood, defined as follows: $\left|N_{\epsilon }(p)\right|$, if $\left|N_{\epsilon }(p)\right|\geq MinPts$, then p is labeled as the core point. If p is in the neighborhood of a core point but does not satisfy the core point condition, then p is labeled as a boundary point.

Clustering procedure: Select a core point p from any unvisited point and create a new cluster C. All points in the neighborhood of p and its ε are added to cluster C. This process can be expressed as follows: $C=\left\{p\right\}\cup N_{\epsilon }(p)$. For each newly added point q if q is also the core point, the neighborhood of q is repeatedly checked, and the cluster C is continued to expand. This process continues until no new core can be found.

Clustering complete: At the end of the process, all the core points and the points in their neighborhood form a cluster, and those that are neither core nor boundary points are labeled as noise.

DBSCAN depends on the distance to define a point’s neighborhood. If the horizontal change is obvious but the vertical change is small, the clustering effect of the points is not ideal. In this study, a 3D digital elevation model is used for clustering. Compared with horizontal distribution information, elevation change information is more effective in accurately identifying deformation regions. Therefore, in the process of three-dimensional spatial clustering, weighted factors should be set to scale the data features of different dimensions and strengthen the attention of spatial clustering.

For the original 3D elevation data, the vertical deformation direction data are weighted based on an unchanged horizontal data scale to enhance the difference of this dimension data. Formula (15) is used to solve the weighting factor w of the vertical data so that the variance of the vertical weighted data is not less than that of the horizontal data.

(15) $$\frac{\sum_{i=1}^{m}{w^{2}\cdot \left(z_{i}-\bar{z}\right)}^{2}}{m}\geq \frac{\sum_{i=1}^{m}{\left(x_{i}-\bar{x}\right)}^{2}+{\left(y_{i}-\bar{y}\right)}^{2}}{m}$$

where $\bar{x},\bar{y},\bar{z}$ are the average values of x axis, y axis, and z axis direction data; m is the total number of sample points of observation data.

## 4. Results and Discussion

### 4.1. Comparison of Three-Dimensional Reconstruction Results

**Figure 11:**
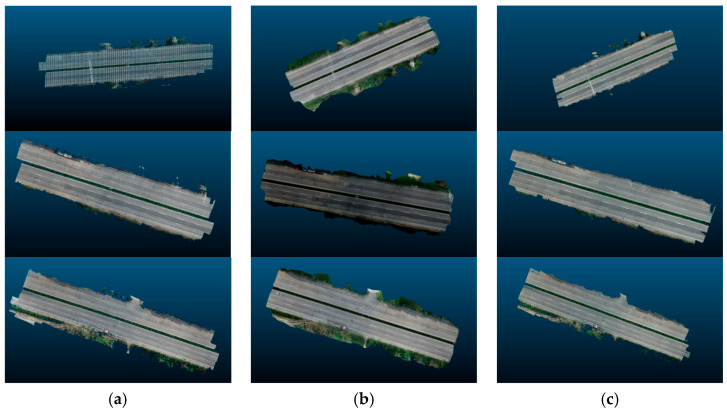
Comparison of reconstruction effect: (**a**) photoscan, (**b**) MVSNet, (**c**) colmap.

After the exit point cloud is reconstructed from the multi-view two-dimensional images of the target pavement scene through MVSNet, we will conduct a comparison experiment between the pavement point cloud obtained in this study and the point cloud reconstructed by traditional colmap and photoscan: the point cloud reconstructed by colmap has higher quality but is slower in terms of reconstruction speed. Although Photoscan is used for three-dimensional pavement reconstruction, the reconstruction speed is fast, but the reconstruction effect is poor, so the detailed comparison experiment between MVSNet and Photoscan reconstruction is not carried out. The evaluation of MVSNet 3D reconstruction is mainly completed through detailed comparison experiments with colmap 3D reconstruction with higher reconstruction accuracy. MVSNet can achieve the reconstruction of the target road scene in ten minutes. In this chapter, the road surface point cloud model reconstructed by MVSNet and the road surface point cloud model reconstructed by the traditional three-dimensional reconstruction method colmap will be compared and tested from two aspects of speed and accuracy under the same hardware environment and the same road surface scene. The results of comparative experiments are analyzed and discussed. Figure 11 illustrates the results of reconstruction using different methods.

#### 4.1.1. Comparison Experiment of 3D Reconstruction Accuracy

In this experiment, the high-precision point cloud model reconstructed by colmap is used as a reference to compare the error between the point cloud reconstructed by MVSNet and colmap. The two groups of point clouds are registered by the ICP (Iterative Closest Point) algorithm. ICP is an iterative process in which registration errors are gradually reduced. To greatly improve the calculation speed, an optimization scheme is used to randomly sub-sample the data clouds at each iteration. The parameter is set to 50,000, which is the maximum number of subsampling points. After registration, the average distance between the two clouds is calculated. The average distance of the point cloud is 0.002849, and the standard deviation is 0.025598 (dimensionless).

Since the calculated average distance of the point cloud is dimensionless, the real-world dimensions corresponding to the point cloud are required for dimensional recovery. Take the coordinates of different feature points in the point cloud, calculate their distance d (dimensionless) in the computer world, and then measure the distance D between the corresponding feature points in the corresponding real world. Multiple groups of d and D can be taken for calculation, and the ratio r between the real scene and the reconstructed point cloud size is 73.1 mm:1, as shown in Formula (16).

(16) $$r=\frac{d}{D}=\frac{1}{73.1\;mm}$$

Therefore, it can be calculated that the average error of point cloud accuracy reconstructed by MVSNet and colmap is 0.57 mm, and the standard deviation is 0.33 mm (as shown in the following Table 3), which indicates that the point cloud accuracy reconstructed by the two is very close.

**Figure 12:**
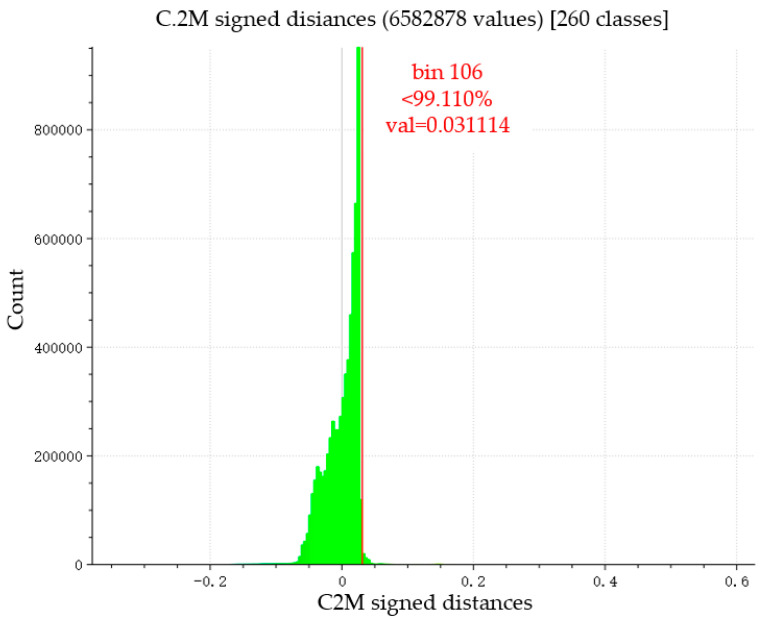
MVSNet reconstructed point cloud compared to colmap reconstructed point cloud distance map.

From the histogram of the distance between two clouds (see Figure 12), it can be seen that the total number of point clouds reconstructed by MVSNet is 6,582,878 points, most of which are distributed in the range of 0 to 0.05 in absolute value.

The results show that the error of the reconstructed point cloud in this study is very small, and the accuracy of the reconstructed point cloud is very similar to that of the reconstructed point cloud in the traditional colmap method.

#### 4.1.2. Comparison of Point Cloud Reconstruction Speed and Density

The evaluation of 3D reconstruction not only needs to evaluate the reconstruction accuracy but also needs to evaluate the time spent on reconstruction.

As for the statistics of the time spent on reconstruction, since the data preprocessing environment of MVSNet reconstruction requires colmap, and MVSNet and colmap have similar processes for data preprocessing, the calculation of the time spent on reconstruction of the two methods does not include the data preprocessing process. Because the resource response allocated by the cloud server may have a certain delay error, the average time of three times of the same reconstruction process is used to calculate the time.

After completing the sparse reconstruction of 178 view pictures of the target road scene and estimating the pose of the corresponding camera, colmap began to carry out the subsequent reconstruction work. This stage included dedistortion, depth estimation, and deep fusion to generate a high-precision point cloud, and the entire reconstruction process took 91 min.

The reconstruction time of MVSNet starts from the input of the processed camera pose and picture and takes 8 min to generate and complete the reconstruction by inputting the picture of the target road scene and the corresponding camera pose information.

The point clouds reconstructed by MVSNet and colmap were respectively intercepted in areas of the same size to calculate the point cloud density. The results are shown in Table 4. The density of the road surface point clouds reconstructed by MVSNet and colmap was 412/cm^2^, and the density of the road surface point clouds reconstructed by colmap was 138/cm^2^. The former was 3.32 times the latter.

This result shows that under the same data set or experimental conditions, the MVSNet model trained in this study has higher reconstruction speed and better accuracy than the traditional reconstruction method colmap and has a significant improvement in the three-dimensional reconstruction of road tables. This technology can quickly complete high-quality 3D reconstruction of pavement, and the reconstruction efficiency is much higher than that of traditional 3D reconstruction technologies such as laser scanning and radar scanning. Compared with these techniques, this study only needs to use multi-view two-dimensional images obtained by ordinary cameras for reconstruction, and the cost is lower. But for complex urban environments, better flight path planning and target recognition algorithms may need to be designed to handle occlusion and other complicating factors.

### 4.2. Test Results of Multi-Section Smoothness and Cause Analysis

In this study, the three-dimensional model of K18+000–K19+000, K19+000–K20+000, K31+000–K32+000, K37+800–K39+000 4 km road section was randomly selected for longitudinal profile observation and flatness analysis. Among them, the longitudinal profiles and elevation values of 1/4 and 3/4 transverse positions of each lane are extracted at fixed intervals and brought into the vehicle model to calculate the smoothness value. According to the Field Test Method of Roadbed and Pavement of Highway Engineering (JTG 3450-2019), the sampling interval of the vehicle laser IRI instrument should be less than 50 cm. In this study, a sampling interval of 25 cm was used to calculate IRI. The IRI of the two profiles on each lane was averaged to indicate the flatness of the entire lane. The following Table 5 shows the smoothness calculation results of the K31+000–K32+000 road section.

**Figure 13:**
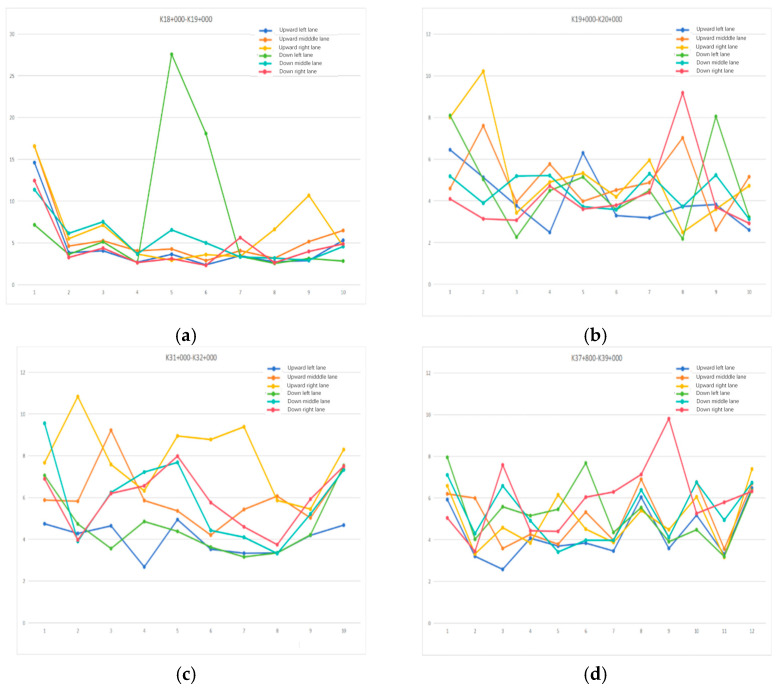
Scatterplot of average IRI values of six lanes on a four-kilometer road: (**a**) K18+000–K19+000 Scatter plot of six-lane average IRI values, (**b**) the K19+000–K20+000 Scatter plot of six-lane average IRI values, (**c**) The K31+000–K32+000 Scatter plot of six-lane average IRI values, (**d**) the K37+000–K39+000 Scatter plot of six-lane average IRI values.

According to the test results of the roughness of the four-kilometer road section, this study draws the scatter plot of the average IRI value of the six-lane road of the four-kilometer road, as shown in Figure 13, to analyze the road roughness in this area. In K18+000–K19+000, nearly half of the IRI values of the down-middle lane and up-right lane are greater than 4, and the road roughness is poor. In K19+000–K20+000, more than 90% of the IRI values are above 4, but most of them are below 8. The upgoing left lane smoothness is relatively good, and the entire smoothness of the kilometer road deviates. In K31+000–K32+000, the overall IRI value of the ascending right lane is larger, and the smoothness is worse than that of other lanes. The ascending left lane and descending left lane have better smoothness, and the overall smoothness of the road in this kilometer is incorrect. In K37+800–K39+000, the trend of IRI values of all lanes is uniform, and most of them are above 4. The upgoing left lane has good smoothness, but according to the trend, it is also gradually deteriorating, and the overall smoothness of the road in this kilometer deviates. Four kilometers of roads were randomly selected for intercept profile observation and roughness analysis, and the results were poor, indicating that the overall roughness of Fuyao Road should be deviated and need to be repaired. Among them, the IRI values of most lanes and road sections are greater than 4. It is recommended to carry out comprehensive road surface repairs, including filling cracks, repairing potholes, and polishing the road surface, and regularly monitor the lanes to ensure that their flatness is maintained in good condition.

### 4.3. Settlement Detection Results and Cluster Analysis

In this study, the digital elevation model of four connected road sections was extracted to identify abnormal deformation of pavement based on DBSCAN clustering, and the clustering accuracy was improved through the data features of weighted pavement elevation dimensions. Table 6 shows the images before and after the clustering of this section. To display the road settlement information more clearly, image processing is carried out on the images after clustering to enhance the settlement characteristics and generate the disease analysis diagram.

According to the road disease analysis chart, the road deformation diseases of this section mainly include rutting, hugging, and local uneven subsidence. Road rutting and hugging are mainly concentrated in road intersections with traffic lights and at the bottom of the downslope, and their distribution locations are mainly located in the middle lane and the side lane near the central divider. The reason for the formation is that there are more heavy-duty vehicles, mainly driving in the middle lane and the side lane of the central divider. Under the repeated action of heavy-duty traffic, rutting and hugging are formed at the wheel track belt. The high-temperature deformation resistance of surface material is insufficient, which leads to rutting more easily. Due to poor bonding between layers, uplift occurs under the lateral action of load.

The irregular settlement of the road section is mainly in the inner two lanes of the road and is often accompanied by mesh cracks. The main causes of the irregular settlement are analyzed as there are more heavy vehicles in the middle lane. The roadbed and soil foundation in the local area of the road are weak, resulting in local subsidence under the repeated action of heavy load traffic, and fatigue cracks caused by the repeated load are not handled in time, resulting in mesh cracks. Local failure occurs in the base layer, resulting in local emptying, and the asphalt surface deformation occurs under the action of load.

## 5. Conclusions and Future Work

This study proposes and validates a 3D reconstruction and large-scale detection method of road surface based on UAV and deep learning technology to improve the efficiency and accuracy of road maintenance. By integrating the improved YOLOv8 model and the MVSNet 3D modeling algorithm, the fast and accurate 3D reconstruction of the road was successfully realized. By introducing point cloud processing extraction technology and the DBSCAN clustering algorithm, the system can effectively identify settlement areas and detect flatness. This method is especially suitable for detecting complex pavement deformation and provides an innovative solution for pavement health assessment.

Compared with traditional vision-based detection methods, the method proposed in this article can effectively improve the accuracy and efficiency of 3D reconstruction through the improved YOLOv8 model and MVSNet 3D reconstruction algorithm. Compared with detection methods based on LiDAR, this method has a lower cost and is suitable for large-scale detection tasks. However, in strong light scenes, the image quality captured by drones may be affected, leading to a decrease in the accuracy of object detection and 3D reconstruction. Meanwhile, when dealing with large-scale data, 3D reconstruction and disease detection still require high computational resources, which will limit the application of this method in real-time detection.

Although the method in this study has achieved good results in reconstruction accuracy and large-scale detection range, there is still room for further optimization. Future research may consider the following directions:

- In practical applications, the accuracy of detection using Unmanned Aerial Vehicles needs to be verified by comparing the detection with the current standard specifications;
- In complex urban environments, especially where there are many buildings and heavy traffic, the difficulty of 3D reconstruction can increase significantly. To improve adaptability in these environments, improved flight path planning, improved image resolution, and enhanced recognition of occluding objects (such as buildings, traffic signs, pedestrians, etc.) may be required. To improve the robustness and generalization of the system, a more diverse set of training data should be used, covering different weather conditions, light environments, and different types of roads;
- Based on the existing DBSCAN clustering, the unsupervised learning algorithm is further optimized to make the system more accurate in the boundary detection of irregular diseases, especially in scenes of complex disease types and fuzzy boundaries;
- Research more efficient algorithm optimization techniques, such as model compression and lightweight network structures, to reduce computational resource consumption and enable detection tasks in complex road scenes.

Through further research and optimization, this method will show higher practicability in the field of road detection and maintenance and provide strong technical support for intelligent road management and maintenance.

## Figures and Tables

**Table 1:** Technical parameters of UAV flight platform.

Camera Parameter	Specifications	Properties
	Sensor	1 in. CMOS
	Resolution	5472 px × 3648 px
	Pixel size	2.4 $\mu_{m}\;$× 2.4 $\mu_{m}$
	Lens	FOV 84° 8.8 mm/24 mm
Aerial flight parameters	Specifications	Properties
	Flight altitude	15 m
	Flight speed	2 m/s
	Course overlap ratio	75%
	Side overlap ratio	75%
	Lens angle	−90°
	Vertical hover accuracy	10 cm
	Horizontal hover accuracy	30 cm
	Ground resolution	0.4 cm/pix

**Table 2:** The division of the road detection area.

Number	Range of Road Piles	Length of Detention
1	K18+000–K19+000	1010 m
2	K19+000–K20+000	1010 m
3	K25+900–K26+700	800 m
4	K31+000–K32+000	1050 m
5	K37+800–K39+000	1212 m

**Table 3:** Error of MVSNet relative to colmap point cloud reconstruction.

Scale	Mean	Distance Standard Deviation
1:73.1 mm	0.000284	0.025598
	0.208261 mm	1.871214 mm

**Table 4:** Comparison of reconstruction effect between the two methods.

Reconstruction Method	Time	Point Cloud Density
MVSNet	8 min	412 units/cm^2^
Colmap	91 min	138 units/cm^2^

**Table 5:** Test results of K31+000-K32+000 road section smoothness.

K31+000–K32+000	Upgoing IRI Value (m/km)			Downgoing IRI Value (m/km)		
Lane Location	Left Lane	Middle Lane	Lane Location	Left Lane	Middle Lane	Lane Location
0–100	4.743	5.8785	0–100	4.743	5.8785	0–100
100–200	4.281	5.824	100–200	4.281	5.824	100–200
200–300	4.645	9.2235	200–300	4.645	9.2235	200–300
300–400	2.6805	5.859	300–400	2.6805	5.859	300–400
400–500	4.9425	5.362	400–500	4.9425	5.362	400–500
500–600	3.527	4.203	500–600	3.527	4.203	500–600
600–700	3.333	5.4305	600–700	3.333	5.4305	600–700
700–800	3.352	6.068	700–800	3.352	6.068	700–800
800–900	4.191	5.0265	800–900	4.191	5.0265	800–900
900–1000	4.68	7.3665	900–1000	4.68	7.3665	900–1000

**Table 6:** Images before and after clustering.

Number	Absolute Elevation Chart	DBSCAN Cluster Diagram	Disease Analysis Chart
Case 1	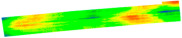	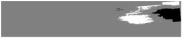	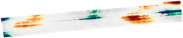
Case 2	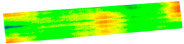	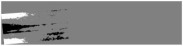	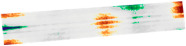
Case 3	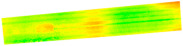	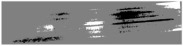	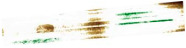
Case 4	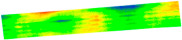	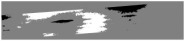	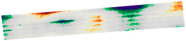

## Data Availability

The original contributions presented in the study are included in the article. Further inquiries can be directed to the corresponding author.

## Abbreviations

1. Abbreviations		
UAV	Unmanned Aerial Vehicle	
MVSNet	Multi-View Stereo Network	
DBSCAN	Density-Based Spatial Clustering of Applications with Noise	
IRI	International Roughness Index	
TP	True Positive	
TN	True Negative	
FP	False Positive	
FN	False Negative	
AP	Average Precision	
mAP	mean Average Precision	
IoU	Intersection over Union	
RANSAC	Random Sample Consensus	
ICP	Iterative Closest Point	
CPU	Central Processing Unit	
GPU	Graphics Processing Unit	
FOV	Field of View	
CMOS	Complementary Metal-Oxide-Semiconductor	
2. Lists of symbols		
**Symbol**	**Unit**	**Mean**
v	m/s	Speed
t	s	Time
d	m	Distance
a	m/s^2^	Acceleration
m	kg	Quality
F	N	Power
π		Pi
e		Base of natural logarithm
f_s_	Hz	Sampling Frequency
A		Matrix
I		Identity matrix
∞		Infinity
d		Differential symbol
∫		Integral symbol
A^T^		Transpose matrix
µ		Coefficient of friction
k		Scale factor
